# Foreign Medico-Psychological Literature

**Published:** 1861-01

**Authors:** 


### FOREIGN MEDICO-PSYCHOLOGICAL LITERATURE.

Our Retrospect of current Foreign Medico-Psychological Literature will embrace the following subjects:—

1. The Meeting of German Alienist Physicians at Eisenach in 1860.
2. Osteomalacia as a Cause of Insanity.
3. The Occurrence of Entozoa in the Insane, particularly the Oxyuris Vermicularis.
4. Hæmatic Swellings of the Ears in the Insane.
5. Congestive Mania.
6. Hysteria and Hysteromania.
7. Hypochondriacal Insanity as a Precursor of General Paralysis.

### 1.—*The Meeting of German Alienist Physicians at Eisenach in 1860.*

Upon the invitation of the Editors of the *Zeitschrift für Psychiatrie*, a number of German physicians, engaged in the management of the insane, met at Eisenach, Sept. 13th and 14th, 1860. Drs. Damerow and Martini were chosen as Presidents for the two days; and one of the subjects which excited a lively discussion was that of "lunatic colonies," introduced by Dr. Flemming. The meeting, while acknowledging how very desirable it is that a portion of the incurable insane should be provided for outside asylums in a suitable manner and at a moderate expense, was most decidedly of opinion that the imitation of the lunatic colony at Gheel, sought for by certain enthusiasts and psychiatrical dilettanti, was not likely to further the interests of either science or humanity. The opinion was very gener..lly expressed that any experiments of this kind should first of all be tried in the vicinity of existing asylums. The further consideration of the subject was adjourned until the next meeting. With respect to the economical care of the insane, the meeting was of opinion that erections are now sometimes made of a needless elegance, and that money is unnecessarily expended upon immaterial portions of these. While admitting the desirableness of entertaining Dr. Schlager's proposition of the preparation of a new legislative code concerning the insane, the meeting deemed it first advisable to examine and arrange all the materials to be found in the various laws and decrees of the different German States. Dr. Jessen besought the Society to use all its influence in order to get psychiatrical chairs and clinics established in all the German Universities. He pointed out the neglect with which psychiatry was treated in these Universities as compared with the other branches of medical knowledge, and the ill consequences which result from the ignorance that prevails amongst practitioners npon the subject, while no ill effect is produced upon the patients in asylums when made the subjects of clinical instruction. Notwithstanding the obstacles which would present themselves to the carrying out this resolution, the meeting felt called upon to adopt it; and, bearing in mind the little zeal which had hitherto been manifested in the study of psychiatry, it also was of opinion that this should be rendered an obligatory part of the curriculum. Dr. Laehr brought under the notice of the meeting the question of the internal arrangements of asylums, as regards water-closets, heating by hot water, and cooking by steam. Those present, who had witnessed the superior construction for these purposes in England, were of opinion that, both as regards material and workmanship, much has to be done in Germany before similar results can be attained. Dr. Ludwig exhibited a specimen of hypertrophic degeneration of one of the olivary bodies, which had occurred in an individual who had exhibited no difficulty in either speech or articulation; the case, therefore, not confirming Schroeder van der Kolk's view of the agency of the olivary bodies in the production of the voice. The proceedings were closed by the announcement that the Editors of the *Zeitschrift für Psychiatrie* offered a prize of twenty gold Fredericks (about 16*l.*) for the best essay upon a subject relating to Psychiatry. Competitors may forwards their essays, observing the usual conditions with respect to mottoes, &c., to the Editors of that journal prior to the 1st of August, 1861.

2.—*On Osteomalacia as a Cause of Insanity.* By Dr. FINKELNBERG.

DR. FINKELNBERG observes that the etiological relation of rachitic softening of the skeleton in childhood to the production of insanity in after years is generally known, and can be illustrated in every large asylum. But whether the nearly allied condition of osteomalacia in the adult, especially as seen in puerperal women, presents an analogous relationship to disturbances of the cerebral functions, has not been noticed by writers on insanity, while it is denied by distinguished writers on female pathology, such as Scanzoni and Kilian. The author in the present paper, communicates two cases in which the first commencement of the psychical disturbance coincided with the acute stage of osteomalacia, no other cause of the condition, which terminated in that of confirmed and incurable insanity, being detectible. Of course, both patients being still alive, the precise nature of any changes which may have taken place within the cranium cannot be stated. From the examination which could be made, however, and guided by what has been recorded by Lucæ and Isenflamm concerning examinations of the cranium in their cases of patients dying with marked osteomalacia (in these instances, however, without insanity being endured) the author supposes that the base of the skull may have participated in the softening process leading to an encroachment upon the cavity of the cranium by the basilar process of the occipital bone, while the walls of the cranium itself have probably undergone considerable thickening and separation. At all events he is desirous of calling the attention of the profession to the probable connexion that may sometimes exist between osteomalacia and insanity.—*Allg. Zeitschrift für Psychiatrie*, Band xvii. s. 199—209.

---

3. *On the occurrence of Entozoa in the Insane, particularly with respect to the Oxyuris Vermicularis.* By Dr. VIX.

THIS monograph, extending over some 160 pages and terminating with 84 conclusions, is occupied far more with the zoological than with the medical relations of the subject. The author's attention was first called to the matter, after having long observed the frequency with which worms are found in the intestines of insane persons dying of marasmus, by a very marked case which occurred in the asylum to which he is attached. The large intestine was found to be lined throughout its entire extent with *oxyures*, which almost entirely constituted the entire mass of the fæcal balls which the intestine contained, the cæcum also containing numbers of *trichocephali.* In the absence of disease in any other organ, the author attributes the symptoms observed during life and the issue of the case to the presence of this immense collection of worms. He adverts, too, to the well-known facts of verminous irritation giving rise to various affections of the nervous system, frequently operating through sympathetic action set up in the genitals. His essay, as we have said, does not contain much of interest to the medical reader. He lays much stress upon the ease and frequency with which the ova may be detected by means of the microscopic examination of the intestinal mucus, and he considers that injections of vinegar, or solution of potass, or of medicinal soap, constitute the best means of destroying the worms, and preventing the development of their ova. — *Allg. Zeitschrift für Psychiatrie*, Band xvii. s. 1, 14, und 227.

---

4. *On the Hæmatic Swelling of the Ears of the Insane.* By Dr. Gudden.

Dr. Gudden first points out that ears closely resembling those of the insane affected with blood swelling are not unfrequently met with amongst ancient sculptures depicting pugilistic athletæ. This condition of the ear was produced by the blow given by the strong arm of the boxer, his hand being enveloped by a leather thong. He next criticises the explanations of their occurrence in the insane usually given, as exemplified in Fischer's treatise, one of the latest on the subject. Fischer believes that such swellings are never met with, unless preceded by chronic inflammation of the cartilage and its covering, the anatomical effects of which consist chiefly in the production of cavities of various sizes in the substance of the cartilage, or between it and the perichondrium. These, the author maintains, are due to the employment of violence, and according to the degree of this, sanguineous effusion also may or may not take place—any inflammation which may result being only secondary to such violence. He also disputes the statement made by Fischer and other authors that these effusions take place only under the influence of scorbutic or other dyscrases or cachexiæ. The only influence of such conditions would be to facilitate the effusion of the blood after the violence.

Among the proofs which he adduces of violence being the cause are the fact of marks of finger-nails being frequently present, the left ear, (more exposed to violence from the right hand) being usually the one affected, and the greater frequency of the occurrence in men than in women; the latter being less exposed to violence and their ears being better protected by their hair and by their dress. Paralytic patients (paralytic males being more frequent than females) are more liable to such swellings than others, not owing to the presence of a dyscrasis, but from their greater liability to ill-usage and their insensibility to its effects. As to the assertion made by Flemming that this injury is self-inflicted, the author replies that during ten years' residence in large asylums he has never met with an instance in which the injury could be traced either to the patient himself or to other patients, while he has been often able to bring it home to the attendants. The frequent accompaniment of the marks of finger-nails show that the ear has been seized and violently pulled by the fingers. Since he has laid down the rule in the asylum to which he is attached of holding the attendants liable for the occurrence of the swelling, the number of cases has remarkably diminished.—*Allg. Zeitsch. für Psychiatrie*, Band xvii. ss. 121-138.

5.—*On a Form of Insanity for which the name of Congestive Mania has been proposed.* By J. H. Worthington, M.D., Superintendent of Friends' Asylum for the Insane, Philadelphia, Pa.

After alluding to the probability of an increase of insanity in a proportion greater than that of population, and to the increase of the severer forms of the disorder, Dr. Worthington proceeds :—

"It is to be hoped so extensive a prevalence of the worst grades of insanity, culminating in general paralysis, as exists at this time in the hospitals of Europe, may never be witnessed in this country. There is, however, a form of cerebral disorder presenting some of the prominent mental characteristics of general paralysis, and arising from the same causes, which, it appears to me, is on the increase in our large cities, and which I have thought might profitably claim the attention of the Association.

"The mental disorder in this class of cases, of which I propose to give a brief description, I have always been in the habit of considering as symptomatic of some grave organic lesion of the brain. From the commencement of the attack, the intellectual disorder is strikingly different from that which is manifested in ordinary insanity. It may appear under any of the usual forms of insanity. The patient may be excited, as in mania and monomania, or depressed, as in melancholia; but, in addition to emotional disorder and the delusions which are prominent characters of these forms of insanity, there are evidences of decided intellectual impairment. The memory is, I believe, nearly always more or less affected, sometimes to the extent of completely blotting out every event of the past life. The patient is generally unable to note the lapse of time, or to form a correct idea of his locality, or of the circumstances by which he is surrounded. Persons affected with this form of insanity are frequently in error respecting their place of abode;—if in a public institution fancying that they are in a hotel, and that they have business requiring attention in the next street. The merchant has some important engagement, the physician his patients whom he is anxious to visit, and the mechanic imagines he has been engaged in his daily occupation, and wishes to return to his family who are expecting him. In these cases the memory, if not entirely null, is so far impaired that the patient is unable to connect his present with his former situation by an intervening chain of events, by which means his erroneous conceptions might be corrected.

"The degree of mental impairment which always exists in these cases, indicating a serious lesion of the cerebral structure, and the consequent gravity of the prognosis, seems to require that they should be distinguished from cases of simple insanity, in which the mental manifestations and termination are so different. The mental condition peculiar to them is scarcely ever observed to originate during the progress of ordinary mania or melancholia. On the other hand, their characteristic physiognomy is strongly impressed upon them from the beginning; so that you will be able to say, with great certainty, that a case is incurable, when otherwise the recent origin of the disorder would warrant the strongest expectations of recovery.

"In some of these cases the insanity, consisting mainly of the most extravagant delusions respecting the wealth or social position of the patient, very closely resembles that form of mental aberration which was until recently considered as almost exclusively belonging to general paralysis. A distinguished French alienist* has not hesitated to class these with the last named disease, even before the appearance of the slightest symptom of paralysis. Another celebrated authority,† while recognising the serious character of these cases, and believing that they frequently end in paralytic insanity, is still unwilling that they should be distinguished from cases of simple insanity, until evidences of impaired muscular action are unequivocally present. A third equally eminent name‡ has declared in favour of separating these cases from simple insanity, on the one hand, and from general paralysis on the other, and making of them a distinct class, under the name of "congestive mania." This term seems well adapted to express the character of the cases which I propose to describe, and I make use of it rather as a matter of convenience than for the purpose of dignifying them with the rank of a distinct disease.

"The prominent mental characteristic of congestive mania is diminished power, which is manifested chiefly by confusion of ideas, incoherence of language, and impaired memory. The term confusion of ideas seems to me very expressive of that condition of mental chaos in which—

Congestaque eodem
Non bene junctarum discordia semina rerum; in which the discordant elements of thought are so confused and mingled together, that the patient is unable to arrange them in an orderly and connected manner. The incoherence differs from that observed in ordinary mania, which results from an exuberance of ideas struggling, as it were, for expression, and forcing themselves into utterance without any regard to orderly arrangement; while in this form of mania, the want of coherence is owing rather to the absence of that mental vigour which is necessary for following out a connected train of thought.

"Failure of memory is, however, the most striking indication of the intellectual impairment. It is not unusual, when patients affected with this form of insanity are taken to a public institution, for them to retain but very indistinct recollections of their journey, and these even may be very soon entirely obliterated. When left by their friends they scarcely inquire after them, or realize the novelty of their position. They imagine themselves to be engaged about their customary business, because they are unable to draw correct conclusions respecting their position from the circumstances in which they are placed, and because their memory fails to present to their minds the succession of occurrences which is necessary to connect, and at the

* Dr. J. Farlet, *Annales Médico-Psychologiques*, tome v. p. 127.

† Dr. Parchappe, *Annales Médico-Psychologiques*, tome v. p. 479.

‡ Dr. Baillarger, *Annales Médico-Psychologiques*, tome iv. p. 579.

same time to separate, their past and present. Patients affected with simple insanity, when placed for medical care in an institution, generally recognise at once the character of the establishment, and frequently manifest considerable ingenuity in framing reasons for their confinement, which may appear to themselves or others consistent with the theory of their own mental integrity. The profound mental impairment of those affected with congestive mania is shown, on the other hand, by the fact, that they are seldom aware of the nature of the institution in which they may be temporarily residing, and if they are partially conscious at intervals of their confinement, they can discover nothing in the fact that is inconsistent with the ideas they entertain of their perfect mental and physical health, or of their exalted station and influence, and never seem to feel the necessity of doing away with the imputation of insanity by explaining why they, who are perfectly sane, should be placed in confinement with lunatics. Those affected with simple madness often display great energy and perseverance in the pursuit of an object, and are very ingenious in adapting their means to the end in view. In congestive mania, on the other hand, patients seldom manifest much perseverance in the accomplishment of their designs, and when they do so the means they employ are often ludicrously disproportionate to the results they anticipate. Sometimes, as in ordinary mania, patients manifest considerable muscular energy and activity, and a strong desire to be in motion, but their activity is generally without an object, and appears to be mechanical rather than voluntary.

"The form which the mental disorder assumes in congestive mania varies in different cases, and is dependent upon the predominance of emotional excitement on the one hand, or of depression on the other. The patient sometimes presents the wild excitement of the highest grades of ordinary mania. He may be violent, destructive, and noisy, and as he is, in consequence of the impairment of his reasoning powers, incapable of being influenced by any appeal to his better judgment, he is frequently very difficult to control. In many cases the emotional condition partakes of that gay and expansive character which has been so frequently described as a symptom of general paralysis. The patient is pleased with himself and every one with whom he comes in contact. He entertains the most extravagant delusions respecting his fortune, his social position, or his personal influence. He believes himself the possessor of immense wealth, and has offices, gifts, or preferments to bestow upon all. He forms the most magnificent schemes for his own and the aggrandizement of his friends, and is most profuse in his promises to those whom he is desirous of enlisting in his service. He thinks himself in perfect physical health, and possessed of great muscular strength, and distinguished mental abilities. These patients are frequently subject to hallucinations, especially of hearing, and voices at enormous distances, which no other ear can hear, are plainly audible to them; and they thus hold conversations with the Almighty, or with distant or departed friends. Their manner is frank, open, and free, and their whole figure and expression manifest the highest degree of satisfaction, contentment, and happiness.

"The prominent delusions, on the other hand, may be of a painful character. The patient will be impressed with the conviction that his sins have incurred the Divine displeasure, and that he can never obtain forgiveness. They sometimes accuse themselves of great crimes, which they say they have secretly committed, and believe that their malady, of which they are to some extent conscious, is sent as a judgment from Heaven to punish them. They believe that their misdeeds have brought extreme distress upon their families, and all that are most dear to them; or that they have rendered themselves amenable to justice, and that the institution where they may have been placed for medical treatment is a prison where they have been sent for punishment, and where they are doomed to undergo the most dreadful tortures. They imagine that they are to be burned or flayed alive; that they are to be scalded to death; that they are to be shot, or hanged, or poisoned. They are frequently harassed by hallucinations, and fancy that they hear voices threatening them with punishment, or devising means for their torture. In some instances they voluntarily seek death, as the only mode of escape from their sufferings; in others, under the impression that it is sinful to eat, or because God has forbidden them to do so, they refuse nourishment for long periods, and in consequence become extremely weak and emaciated. They frequently imagine themselves to be the victims of some secret persecution, and that their enemies are seeking means to compass their destruction. They believe themselves acted on by some mysterious influence which they call magnetism or electricity, and by which they suppose their enemies are able to injure them without fear of discovery.

"In another class of cases the emotional disturbance may be very slight, even at the commencement of the attack, and there may be very little outward manifestation, either in language or conduct, of the serious nature of the disease, which may have fastened itself irremediably upon the patient. He may quietly entertain some delusion respecting his fortune or social position, or believe himself under the special guidance and protection of the Almighty, and may be subject to various hallucinations, while his language and deportment, to common observation, may be those of a sane person. But in these cases there is always marked impairment of the mental faculties, under the form of enfeebled memory, or inability to comprehend any subject upon which you may wish to fix his attention. When conversing with a case of this description, you will sometimes be made painfully sensible of the futility of every effort to impress him with a new idea, while he may perhaps talk sensibly and rationally on subjects with which he is already familiar.

"The above are the prominent mental characteristics of the cases of insanity which I propose to describe under the name of congestive mania. The most of the symptoms which have been named are, however, met with in cases of simple insanity, under one or another of its various forms, and I would now call the attention to a different class of symptoms, which may be considered as peculiar to the congestive form of the disease, and therefore as distinguishing it from simple insanity. These are the physical phenomena indicating the congestive character of the disease, which has attacked the nervous centres.

"Among the general symptoms peculiar to congestive mania, are those which indicate cerebral oppression; and these may vary from slight giddiness or confusion of ideas, to the most complete deprivation of sense and motion. Instead of the heightened sensibility to external impressions, which is a striking characteristic of simple mania, there is always in the congestive form diminished acuteness of perception. Though the organs of the special senses may be perfect, the brain seems incapable of receiving clear and distinct impressions of outward objects, so that the patient rarely forms correct ideas of the circumstances in the midst of which he is placed. As in general paralysis, there is diminished sensibility to pain, and in some instances, where the congestion extends to the portions of the brain supplying nerves to the sensitive organs, there is impairment of vision or of the senses of smell and taste, and patients sometimes experience a sensation of numbness in the extremities. Another set of symptoms which indicate cerebral congestion, are those which, without amounting to paralysis, are yet evidences of diminished muscular power; such as tremulousness of the hands, lips, or tongue, unequal dilatation of the pupils, and indistinct articulation, when it is slight, and when it is observed only at long intervals. In some cases the whole muscular system seems to be remarkably deficient in energy. The patient walks bending forward, or with a shuffling motion of the feet, and all his movements are stiff and constrained; or he reels from side to side in walking, like an intoxicated person. In other cases there is evident though slight paralysis, which is frequently temporary, confined to a single muscle or set of muscles, and manifested by the drooping of an eyelid or slight relaxation of the muscles of one side of the face. There are cases, again, where the muscular system is affected with spasm—there may be grinding of the teeth, or muscular jerkings of the extremities, or the whole system may be affected with convulsions which closely resemble those of epilepsy.

"In some cases the disease commences with an attack of cerebral congestion, during which the patient remains unconscious for perhaps only a short period. On recovering consciousness he will appear confused and bewildered, and the mental disorder will gradually increase until it amounts to decided insanity. At the same time the pupils may be unequally dilated, or muscular tremors may be observed in the tongue or lips, or in the upper extremities. In other cases there may be several attacks of unconsciousness, without any appearance of mental aberration for a considerable time. In others, again, the congestion may be so slight as not to render the patient unconscious, and he will complain only of giddiness and confusion of ideas, until at length mental disorder will become more manifest.

"In another class of cases the mental aberration exists for a considerable time before the physical symptoms, indicating the nature of the disease, make their appearance. Here, however, the insanity generally manifests that peculiar tendency to dementia, which has been mentioned above as denoting the congestive form of the disease.

After the attack has existed for several weeks or months, the patient will be found exhibiting symptoms of decided cerebral congestion. Having previously been in a condition of high mental excitement, he will all at once appear silent, subdued, and bewildered, he will be unable to comprehend anything that is said to him, will perhaps be unable to speak, and in walking his body will incline to one side. These symptoms may continue for a few hours, and under appropriate treatment the patient may gradually be restored to his former condition, or they may speedily be followed by an attack of convulsions resembling epilepsy, succeeded by coma of many hours' duration.

"Some of these cases are presented under the sub-acute form, and resemble somewhat that affection which has been described by Dr. Bell of Massachusetts as a new form of disease, by different authors under the name of acute delirium, and by Dr. Calmeil as insidious peri-encephalitis. Patients manifesting the symptoms which have been described under the above names, sometimes linger a considerable time, and before death, and even during the whole progress of their disease, present tremors and other signs of muscular impairment, which have been described above as peculiar to this form of disease, and as indicative of cerebral congestion. In these cases the patient sleeps but little, and the digestive functions become seriously implicated. The natural desire for food and drinks is entirely lost, the tongue is covered with a thick fur, and at length becomes dry and brown, the breath has a peculiar acid or an offensive odour, emaciation rapidly progresses, eschars form on the parts of the body most subject to pressure, and the patient dies, apparently exhausted in consequence of long continued nervous irritation, and impairment of the nutritive functions, rather than from the direct action of the disease upon the brain itself.

"In congestive mania there is a strong tendency of the system to that form of general, functional impairment, which has been described by Dr. Parchappe under the name of *cerebral marasmus*. Under the influence of the various painful delusions from which these patients so frequently suffer, but more especially owing to the depressing effect of the cerebral disorder upon all the functions, the vital powers become gradually exhausted. Sometimes, even when food is taken freely and regularly, the patient rapidly emaciates, and his muscular strength diminishes, until he is no longer able to keep on his feet. In these cases of marasmus there is frequently a tendency to the formation of abscesses in the subcutaneous cellular tissue, which may thus become infiltrated with pus in large quantities. The mucous membranes appear to be particularly disposed to take on inflammation, and troublesome diarrhœa or bronchitis frequently sets in. The circulation gradually becomes weaker, and eschars form over the sacrum or trochanter. All these complications tend to exhaust the remaining strength of the patient, and to hasten the fatal termination of the disease.

"In some cases where the patient has remained stationary for a considerable time, enjoying a good share of bodily health, symptoms of acute cerebral disease will be all at once presented. The pulse will become frequent, there will be almost entire absence of sleep, the delusions will be of the most painful and distressing character, the patient will be in constant agitation, and will require to be kept in bed by main force, and will obstinately refuse food and medicine and every attention that his case requires. With these symptoms he will go on from bad to worse for several days, when he will suddenly be found in a sinking condition, and die in a few hours in a state of profound collapse. In other cases a comatose condition, sometimes preceded by convulsions, at others becoming more gradually established, makes its appearance, and is followed by death in a few hours.

"Some cases occurring in young persons of good constitutions, who have presented the symptoms indicating a slight degree of cerebral congestion, are gradually restored to mental health and vigour. In other cases, though the delusions and all emotional disturbances have vanished, and there is no decided symptom of mental disorder remaining, there is something about the patient which leaves a doubt on the mind of the physician of his entire recovery. In these cases there is a slowness and manifest effort in the intellectual operations, which clearly show the injury which the organ of thought has sustained, and its consequent unfitness for performing its functions with the quickness and ease natural to it in health. Such patients are liable to a renewal of the original cerebral disorder, which may be of so aggravated a character as speedily to destroy life, or the brain may only be injured to an extent which leaves the patient permanently demented. In some cases the disease, after a course of many months, takes on the characters of general paralysis. The patient gradually loses the power of articulation, his gait becomes unsteady, his limbs at length refuse to support his weight, and all the symptoms of that form of cerebral disease are fully established.

"The character of the mental aberration—the chaotic confusion of ideas, the incoherence, the impairment or loss of memory, the inability of the patient to form correct conclusions respecting his locality from surrounding objects, all indicating a profound lesion of the intelligence—will in any case of insanity of recent origin be sufficient to arouse the fears of the practical alienist, who will at once conclude that in a case presenting this form of aberration he has to manage a very different disease from ordinary insanity. Dr. Parchappe* ranks all cases presenting this mental condition with simple insanity, and considers them as cases of purely *dynamic* or functional disorder, while he attributes the *plastic* or organic character to those cases only which present the complication of general paralysis. Cases are, however, frequently met with, sometimes under the form of ambitious mania, sometimes under that of melancholia or hypochondriasis, presenting the condition of mental impairment which has been described above, but without the slightest symptom of paralysis, which, on account of their incurable character, or their speedily fatal termination, it is of the utmost importance, in a practical point of view, to distinguish from simple insanity. Dr. Guislain (*sur les Phrenopathies*, vol. i. p. 368,)

* *Annales Médico-Psychologiques*, vol. iv. p. 475.

has spoken of the difficulty of distinguishing insanity accompanied with cerebral congestion, during its early stages, from the simple uncomplicated form of the disease. 'The conditions,' says he, 'which excite the fears of the physician, are the persistence and increase of the mental disorder, the complete absence of moments of calmness and rationality, the appearance of acute symptoms in a case which has become chronic, confusion and incoherence of ideas, accompanied with feebleness of conception and memory, spreading itself like a veil over all the perceptions. * * You may suspect its existence if, from the origin of the malady, you observe violent passions in connexion with great disturbance in the domain of thought, and if you observe ideas which recall a state of marked intoxication, if from the beginning the conversation is incoherent, if the words have neither order nor connexion, and if there is exaggeration, existing at the same time with enfeeblement of thought, if the answers of the patient bear the impress of extravagance, if he boasts with a puerile air of his bravery, his wealth, or his intellect.' This author remarks that as long as the ideas are clear, however extravagant they may be, there is no reason to fear cerebral congestion. It is not to be suspected in simple insanity, or in cases where an exaltation of the passions or emotions, or even an unaccustomed impulse of the will, characterizes the disease, nor in any other form of insanity which does not present indications of decided intellectual impairment. He speaks of emaciation, muscular rigidity, involuntary evacuations, convulsions, and paralysis, as diagnostic signs of this complication. When the congestion produces effusion between the membranes, 'the symptoms,' says he, 'are sometimes truly alarming. They consist of a sudden change in the mental and physical condition of the patient. Sometimes a state of coma is followed by a notable loss in the sum of the intellectual acts, in other cases there is incomplete hemiplegia, muscular contractions, jerkings, or general convulsions, followed by complete suspension of all the sensorial acts.'

"The above symptoms when fully developed will leave no room for doubt as to the nature of the disease, as distinguished from simple insanity. During its forming stage, before the certain indications of congestion have made their appearance, the existence of such evidences of intellectual impairment as have been described will put the physician on his guard against deciding too hastily as to the harmless nature of the malady. From general paralysis, it may be known by the absence of all symptoms of paralysis, except such as are only occasional and temporary, very partial in their extent, and so slight as scarcely to attract notice. In that disease, the paralysis, though slight in the beginning, is manifested in all parts of the muscular system, is constantly progressive, and at length, both in extent and severity, comes to be the most striking feature of the disease.

"That there is a very strong relationship between congestive mania and general paralysis, is fully attested by the resemblance of the mental disorder, and the identity of causes which produce the two forms; and if it were possible for paralytic insanity to exist without paralysis, we might feel tempted to refer both classes of cases to that affection as to an admitted and well-established form of cerebral disease. There is indeed strong ground for believing them to be identical in their nature, and that consequently the paralysis is not an essential feature, but only a complication or one of the modes of termination of the disease. Dr. Parchappe, it is true, classes all cases such as I have been describing with simple insanity, under the head of purely functional disorder, and makes them essentially distinct from cases of general paralysis, which he considers as dependent upon a structural, organic change of the cerebral tissue. I believe, however, that softening, such as has been observed in the cortical substance of the brain, to which he attributes paralytic insanity, is far from being admitted by pathologists as a primary, idiopathic affection. If this softening is not entitled to be so regarded, the paralytic symptoms cannot be properly referred to it as their cause, but to some anterior morbid action of which it is the result. Dr. Calmeil* considers this morbid action to be inflammatory in its nature, and has given it the name of chronic diffused peri-encephalitis. The same author describes cases of the acute cerebral affection which has been already mentioned under the name of acute delirium, as acute peri-encephalitis under the insidious form. The resemblance of congestive mania to that affection has already been spoken of, and it appears to me that it occupies the position of a connecting link between the acute and chronic forms of the same cerebral disease, viz. a congestive or inflammatory affection of the cortical cerebral substance, in its most acute form, running a rapid course, and generally terminating fatally from the eighth to the fourteenth day, sometimes in its milder forms ending in apparent recovery, but frequently passing into the variety of chronic mania which I have been engaged in describing, and sometimes ending in general paralysis. All may not be agreed upon the inflammatory nature of the affection, but the fact that it is constantly accompanied with cerebral congestion will perhaps not be denied by any one, and the word *congestive*, expressing this fact, though possibly not fully indicating the nature of the disease, appears to me to be highly appropriate, as well as convenient, for designating those cases in which the paralytic symptoms are absent. It has been proposed for this purpose by Dr. Baillarger, in whose opinion this form of mania frequently terminates in paralytic dementia, to which it bears the same relation that simple mania does to simple dementia." (*American Journal of Insanity*, Oct. 1860.)

---

### 6.—*Hysteria and Hysteromania.*

An anonymous writer in the *American Journal of Insanity* (October, 1860) introduces a report of several interesting cases of hysteria and hysteromania, by the following remarks :—

"Much has been done to render medical science more simple and precise, in the withdrawal from several classes of disease, which embraced a large number of diverse and obscure symptoms, of special

* *Traité des Maladies inflam du Cerveau*, vol. i, p. 261.

groups of these, dependent upon some pathological or other proximate cause, whence they might be appropriately named. Thus in the progress of science, from the hypothetical disease insanity have been taken the better defined maladies, of mania à potû, general paralysis, and softening of the brain. If there have also been added to insanity a number of special varieties, each representing a single form of moral perversion, this error in classification is likely to be soon abandoned. The same is true of epilepsy, which remains an ideal type of disease, while epileptiform convulsions, frequently found to be dependent upon certain cerebral lesions, toxic agents, or points of irritation, in numerous instances are not included under this name. It would seem expedient to follow a similar method in treating of hysteria, and it is matter of surprise that this has not already been done. We would thus denote by the name hysteria, a disorder marked not only by great nervous excitability, shown in spasm or convulsion, but also a perversion of the emotional nature, with more or less impairment of the voluntary powers. Simple convulsion, even of the kind most common in hysteria, would not then be termed hysterical, unless connected with moral or volitional disturbance, and no disorder of the cerebral functions would be classed under this head, unless conjoined with a morbid state of the spinal nervous system. Thus, in a case of hysteriform convulsions following some sudden sensation, or some new and entirely unsolicited emotion—the primary hysteria of many writers—we would not admit the term. If this exclusion were general, many chaste and right-minded women, who chance to have suffered a peculiar form of convulsion, would escape an unjust imputation; while in many others, whose nervous functions were unimpaired, might be recognised the premonitions of insanity or the proofs of a vicious nature, both of which are too often passed by under the disguise of an unmeaning term. In the so-called secondary hysteria, following upon emotions voluntarily preferred, or upon the spontaneous recurrence of those before indulged, or from sympathy with morbid emotions in others, the disorder should alone be recognised.

"By hysteromania we do not mean a condition of 'moral insanity,' marked by 'irresistible impulses' to the intensest egotism, to mendacity, and sexual gratification, but a true mania, developed upon a state of hysteria, to which it bears the same relation that mania à potû does to habitual drunkenness, the 'oinomania' of certain writers. In confirmed inebriety, as in hysteria, there is a morbidly powerful passion, with which a correspondingly weakened volition has to contend. But principles of conscious right and of the soundest policy, hold the individual responsible for the actions which follow. The first stimulus to the growth of the passion in each case, was, or must be presumed to have been, voluntarily applied, and the loss of the determining power of the will can only be proved by positive symptoms of cerebral disorder. This will no doubt be more readily assented to in reference to drunkenness than to hysteria. In those rare cases of drunkenness, resembling most closely paroxysms of mania, the gratification to be obtained is one in some degree conceivable by nearly all who are called to pass upon the responsibility of the subject. But in the worst forms of hysteria the desire from which all the almost supernatural efforts flow, is utterly foreign and incomprehensible to the common mind.

"The limits of hysteria and hysteromania are then to be determined, as in deciding between confirmed inebriety and mania à potû, by the presence or absence of actual lesion of the mental faculties, implying a coercion, not a surrender or a depravation of the will."

---

7. *Hypochondriacal Insanity as a Precursor of General Paralysis.* By M. Baillarger, of the Salpetrière, Paris.

General paralysis is a common and most serious phase of mental disease. It attacks patients of all ages, and its progress towards a fatal termination exhibits stages of the most melancholy and humiliating nature. All medical men are of accord that it is most insidious in its approach. It may be long in becoming fully developed, presenting at first only the most trivial indications, in many cases so trifling as to pass altogether unobserved; and when the malady does at last attract attention, it may be too late for arresting its advance. It is therefore most important to attend to this disease at the very first; and it is with this object that it seems useful to describe the intimate relation existing between the hypochondriacal form of melancholia and general paralysis.

This relation being understood, it becomes one means of detecting the advent of that disease at the very commencement of its attack. It is of importance to distinguish this symptom, as the melancholy accompanying general paralysis very much resembles melancholia in its simple form. The conceptions or illusions of the hypochondriac, however, although of considerable variety, are yet of such a tendency as often to present something of a special character in their nature. The patients believe that their various organs are changed, destroyed, or completely obstructed: they pretend, for example, to have no mouth, no abdomen, no blood—that their gullet is stopped up, their stomach quite full, their bowels shut up; they imagine that their food passes from its ordinary channels—that it gets into their skin, or even their clothes. Four patients believed their body to have become putrid. Many among these patients are afflicted with hallucinations of the sense of smell. Some keep their eyes closed, and allege that they are blind; others cease to speak, and state afterwards that it was impossible for them to open their mouth; they assert that they cannot either swallow, or defecate, or make water; they affirm that their members are altered—that they are larger or smaller; they say that they do not exist, or even go so far as to believe themselves dead; they remain motionless, the eyes shut and when their limbs are lifted they let them fall, as if completely paralysed. These different delusions lead to serious consequences: many of the patients refuse, more or less obstinately, to take food, and sometimes it becomes necessary to feed them by means of the stomach pump; such patients speedily become much emaciated. I have seen, M. Baillarger states, a lunatic die in eight days from obstinately resisting the employment of the stomach pump, under the impression that his stomach was completely full, and his gullet obstructed. One patient pretended he could not make water, and used every effort to retain it; his bladder became enormously distended, and he was at last attacked by a veritable retention, and it was with great difficulty the catheter could be used. In the end a false passage was made, and the patient died, while yet in the first period of the disease.

The tendency to gangrene, which is one of the characteristics of general paralysis in its latter stages, exists in these cases markedly, and before its usual period. Four cases had large eschars over the sacrum, without ever having been confined to bed; one woman, who for a year had exhibited all the symptoms of commencing general paralysis, preserved every appearance of health in other respects, when, all of a sudden, she became affected with hypochondriacal melancholia, and six weeks afterwards died of gangrene in both feet.

Hypochondriacal delirium is thus not only a mere premonitory symptom of certain forms of paralysis, but it is a serious symptom, and one very unfavourable, in prognosis.

In reference to this affection—viz., that of hypochondriacal insanity—viewed as one of the precursors of general paralysis, this being the fact of most practical value in connection with it, the delusions of which we have spoken seem to claim especial attention, as they are sometimes to be detected in patients as yet evincing no indication of paralysis,—this supervening at a later period. Such a termination is certainly not invariable; but it is so common after this symptom, and the prognosis in such cases is so unfavourable, that considerable importance seems to attach to the subject. Thus Dr. Combes published some remarks on a case of lypemania, with stupor, and other serious symptoms,—nothing, however, indicating that at a later period this patient would be attacked with paralysis; and, after fifteen months' residence in this asylum, where he was treated, he was dismissed as cured. In reading Dr. Combes' remarks, I was struck, observes M. Baillarger, with certain of the delusions affecting this patient. He had believed that he was about to die, if indeed he was not already dead; that his limbs were atrophied, that he had none, &c. These appearing to be good grounds for suspicion, I wrote to Dr. Combes to know what had become of the patient. The answer confirmed my suspicions—the result having been that, after a year's return to his occupations, he had been attacked with general paralysis. We may see by this example, that, had hypochondriacal delirium been held as a certain precursor of general paralysis, this affection might have been foretold two years before it actually took place.

It may appear strange that one form of insanity should thus be urged as premonitory of paralysis. Singular as it may seem, however, it is not the first time that such a doctrine has been urged. Since the writings of Bayle, no medical man doubts the fact of certain forms of insanity, such as the ambitious form, being symptomatic of approaching paralysis. And if one form of delirium be held, in mania or monomania, as indicating the advent of paralysis, there seems no reason why this particular hypochondriacal form should not serve the same purpose, and with equal certainty, in melancholia.

We do not attempt to explain these facts; and we may add, it seems useless to do so, either here or in the case of ambitious insanity. One point connected with the ambitious form may be mentioned; and that is, the relative frequency of general paralysis among females in different ranks of society. While this malady is equally common among males of all classes, among women it is not so. It is very common among the poor, and very rare among the rich. It would appear, however, that this circumstance has been forgotten by those who would explain the greater number of cases of ambitious insanity as induced by ideas of speculation—by the desire of suddenly arriving at honours and fortune.

In conclusion, it appears evident that hypochondriacal no less than ambitious insanity may, in different circumstances, be considered as a prognostic of general paralysis. The intention of the present paper has accordingly been to direct attention more particularly to the latter of these forms. As for the first, I have frequently had occasion, before now, to refer to it in all its remarkable psychological characters. —*Annales Médico-Psychologiques*, Oct. 1860.

